# Interprofessional Collaborative Clinical Practice in Medicine and Pharmacy: Measure of Student Perceptions Using the SPICE-R2F Instrument to Bridge Health-Care Policy and Education in France

**DOI:** 10.3390/healthcare10081531

**Published:** 2022-08-13

**Authors:** Alexandre Piogé, Joseph Zorek, Jens Eickhoff, Blaise Debien, Julie Finkel, Alexandre Trouillard, Patrick Poucheret

**Affiliations:** 1 Centre de Formation et de Recherche en Pédagogie des Sciences de la Santé, Université de Strasbourg, 67000 Strasbourg, France; 2 Qualisud, Université de Montpellier, Université d’Avignon, CIRAD, Institut Agro, IRD, Université de La Réunion, 34000 Montpellier, France; 3 School of Nursing, University of Texas Health Science Center at San Antonio, Linking Interprofessional Networks for Collaboration (LINC), Office of the Vice President for Academic, Faculty & Student Affairs, San Antonio, TX 78229, USA; 4 School of Nursing, University of Texas Health Science Center at San Antonio, San Antonio, TX 78229, USA; 5 School of Medicine and Public Health, University of Wisconsin-Madison, Madison, WI 53705, USA; 6 Centre d’Enseignement de Soins d’Urgence, Centre Hospitalo-Universitaire de Montpellier, 34000 Montpellier, France; 7 Département de Pharmacie, Centre Hospitalier Universitaire de Nîmes, 30000 Nîmes, France

**Keywords:** interprofessional collaborative clinical practice, medicine, pharmacy, health care, SPICE-R2F, pedagogy, curriculum, France

## Abstract

Background: Public health policies in France and the USA promote health professionals’ collaborative practices in accordance with World Health Organization recommendations emphasizing the need to promote interprofessional education and training. To optimize alignment of health-care policy and education, a scientific evidence-based approach is required. Methods: A French translation (SPICE-R2F) of the Student Perceptions of Interprofessional Clinical Education—Revised instrument, version 2 (SPICE-R2) was generated. SPICE-R2F was then completed by a multicentric cohort of French health students, and confirmatory factor analysis was utilized to evaluate the validity and reliability of this instrument based on response patterns. Results: Translation of SPICE-R2 was validated evaluating psychometric properties and conducting a confirmatory factor analysis (CFA). Adequate model fit was demonstrated using RMSEA (root mean square error of approximation) and CFI (comparative fit index) model fit criteria. Within each factor, however, low to moderate levels of reliability were observed between items. These observations diverge from other countries and highlight a potential French singularity. Conclusion: Our results suggest the need to improve interprofessional clinical practice education in France at early stages in the health-care curricula. The SPICE-R2F instrument may represent a valuable evidence-based tool to characterize perceptions of interprofessional education and training of health-care students and professionals in France.

## 1. Introduction

In France since 2003, public health policy has advocated for collaboration among health professionals. The French High Authority for Health (HAS) therefore recommended in 2008 new forms of cooperation including task delegation associated with the emergence of potential new health-related professions [1]. In 2009, the French law titled “Hospital, patients, health and territories” (HPST) formalized the concept of cooperation with article 51 specifying: “By way of derogation, health professionals (cited in Article L. 4011-1 of the CSP) may engage, on their own initiative, in a cooperation approach aimed at carrying out between them transfers of activities or acts of care or to reorganize their mode of intervention with the patient” [2]. This law represented a legislative starting point for health-care interprofessional collaborative practice (IPCP). HAS stressed that cooperation is to be seen from the standpoint of procedure substitution [3]. As paragraph number 9 of the 2016 national Great Health Conference stated: “*The division of labor within the health professions is progressing inexorably. […] it is driven by technological developments, specialization, even hyper-specialization of training, pricing practices. […] The care pathway for patients with chronic diseases is characteristic of these developments: on the one hand, interventions are increasingly diversified with people throughout the care chain […]; on the other hand, this diversity calls for a need for integration, cooperation, coordination so as not to leave it up to patients to manage the dispersion. Deploying a common training base will help improve the inter-knowledge of professionals essential to the development of cooperation*” [4]. Following these institutional recommendations, several examples of IPCP emerged. More specifically, multidisciplinary health centers (MSPs, i.e., “*Maisons de santé Pluriprofessionnelles*”) for primary care were created in France. These practice models aimed to consolidate resources and advocated for interprofessional activities, including medical, paramedical, and pharmaceutical professions. An illustration of this institutional trend was the national public health plan titled “*Ma santé 2022*” (i.e., “*My Health 2022*”), reinforced by the conclusions of the “*Ségur de la santé*” (i.e., national mission of consultation for health). One of its objectives was to double the number of MSPs in 2022 [5]. However, if these practice models promote health-care providers’ collaboration, are they sufficient to set up a national interprofessional collaborative clinical practice?

At the international level, the World Health Organization defines IPCP as follows: “*When multiple health workers from different professional backgrounds work together with patients, families, health care professional and communities to deliver the highest quality of care*” [6]. This definition does not evoke a substitution of tasks, but work in common among health professionals, patients, families, and communities. It recognizes that interprofessional practice strengthens health systems and improves health expectations. The WHO explained in its 2010 framework that the implementation of interprofessional education (IPE)—a pedagogical approach to train future and current practitioners in the requisite attitudes, perceptions, knowledge, and skills in this area—promotes collaboration between health professionals in practice [6]. Since 2010, numerous experiments, predominantly in North American and European English-speaking countries, have established the value of IPE [7] and its integration into health curricula [8]. A model developed by the US-based Institute of Medicine, the interprofessional learning continuum model (IPLC), emphasizes the importance of data collection in the initial phases of curricula, especially from the very first years, to monitor and manage IPE implementation and resulting learning outcomes (e.g., changes in perception, knowledge, skills, and behaviors) [9]. Nowadays, IPE for pharmacy and medicine students is mandatory in the USA. This is illustrated by the national consensus guidance published in 2019 by the Health Professions Accreditors Collaborative (HPAC), a group comprised of 25 health professions’ accrediting bodies charged with regulating the quality of educational programs in the US [10]. The pharmacy and medicine accrediting bodies enabled integration of IPE into the accreditation standards for pharmacy [11] and medicine doctorate degrees [12]. Therefore, data collection and analysis should be considered a foundational element in the development of IPE and IPCP programs.

Various psychometric tools have been developed and described in the literature to measure perceptions of health professionals and students regarding interprofessional learning and teamwork. A first example is the Readiness for Interprofessional Learning Scale (RIPLS), which has been translated in French [13]. This tool has been questioned on several aspects of its initial methodology and grid of evaluation [14]. A second example is the Student Perceptions of Physician–Pharmacist Interprofessional Clinical Education instrument, first published in 2013 [15]. This tool, focused on medicine and pharmacy learners, is practical and efficient to evaluate perception and conceptualization of IPE in pharmacy and medicine students. The instrument is based on the competence framework of collaborative interprofessional education [16]. It has been translated into several languages. For instance, in Europe, the instrument was translated and validated in a German-speaking student population [17]. Recognized for its psychometric reliability and its ability to assess student perceptions, this instrument was used to evaluate interprofessional training and educational outcomes associated with IPE to support health-care system transformation [18].

The rationale for our research program lies in the fact that in France, when compared to others countries, IPE and IPCP are still at an experimental stage. It therefore allows the development of an appropriate foundation to contribute toward reaching the objectives defined by French public health policies about interprofessional education and collaborative practice.

The scientific question to be explored is: How and to what extent can we implement interprofessional training in France, a sine qua non for the emergence of health interprofessional collaborative education and practice?

The objectives of the present study were: (i) to validate a French translation of the SPICE-R2 instrument (SPICE-R2F), (ii) to define the actual status of IPE and IPCP perception in two multicentric cohorts of French students, one in pharmacy doctorate and one in medicine doctorate courses, and (iii) to make available the psychometrically validated SPICE-R2F version to the larger French-speaking health-care scientific educational community to manage IPE and IPCP development. To our knowledge, this is the first time that such an investigation and scientific approach using SPICE-R2F has been undertaken to support and consolidate interprofessional collaborative education and training of health-care students and professionals in France.

## 2. Materials and Methods

### 2.1. Translation Process

**Figure 1:**
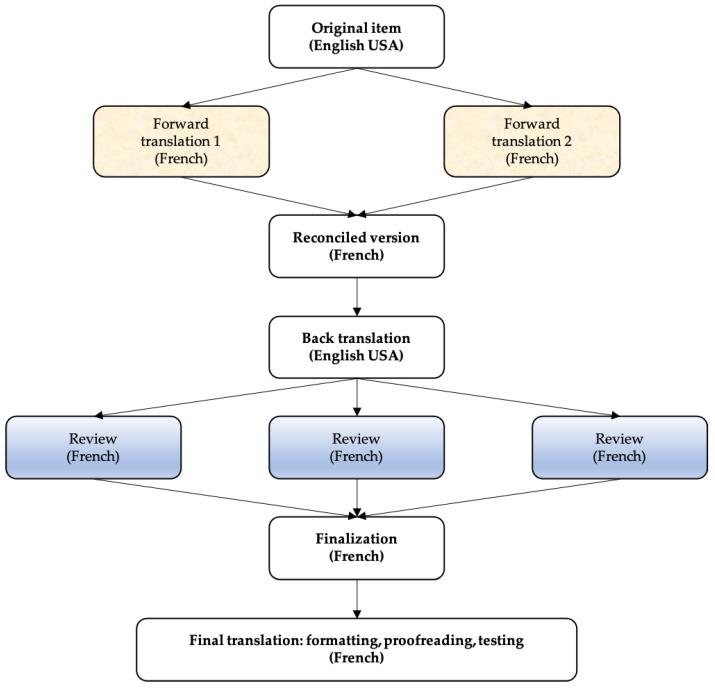
FACIT method.

We followed the FACIT (Functional Assessment of Chronic Illness Therapy) method described by Eremenco et al. (Figure 1) [19]. The original tool consisted of a three-factor structure with 10 items, each measured on a 5-point Likert scale. The three factors (subscales) were defined as follows: the measurement of students’ perceptions of interprofessional teamwork and team practice (factor 1), the roles/responsibilities of collaborative practice (factor 2) and patient outcomes of collaborative practice (factor 3). Total and subscale scores were calculated as the sum over all item scores. The instrument was translated into two separate versions by two English speakers. We then obtained a consensus on the two versions and wrote a first translation. This consultation was done in exchange with third parties, also English-speaking. We then assessed the robustness of our work by back-translating from French to English. This step was performed with two English-language professionals (university and language translator). We were able to finalize the French translation by testing it with pharmacy students and colleagues (physicians and pharmacists). The French version (SPICE-R2F) of the instrument was generated.

### 2.2. Cohorts

For France, our data collection favored a multicentric approach, by testing the translation of the instrument on the promotions of second-, third-, fourth-, and fifth-year medicine and pharmacy students from the universities of Bordeaux, Montpellier, Paris, and Strasbourg. For each university, a request to participate was made to the faculties (UFR, i.e., Unit of Formation and Research) of medicine and/or pharmacy. These two types of students are rarely brought together during their education and training. Participation in the project involved sending an email with the internet link to the instrument. The latter was hosted on the LimeSurvey platform in the form of a questionnaire. For each of the 10 questions, the perceptions of the students were gauged using a 5-level Likert scale ranging from “strongly disagree” (1) to “strongly agree” (5). A “neutral” option was included. The link first led to a description of the study and a consent form. Agreement via the consent form was mandatory before being able to access the instrument. For all the UFRs, the students received only one request to complete the questionnaire. Access to students’ emails, demographic data, and sector and year of study were collected and managed by the administration of the UFRs, where the students participated in the study.

### 2.3. Statistical Analysis

Statistical analyses were performed on SAS (SAS Institute, Cary, NC, USA), version 9.4, and MPLUS (Muthen and Muthen, Los Angeles, CA, USA), version 8.4. Due to the observational nature of the study design, no formal sample-size calculation was conducted. Instead, the sample size was based on feasibility considerations and to provide a sufficient level of robustness in parameter estimation. The demographic information of the participants, French students in medicine and pharmacy, was characterized using descriptive statistics. The validity of the SPICE-2RF instrument was evaluated using confirmatory factor analysis (CFA). Parameter estimation was conducted using the maximum-likelihood method. Standardized factor loadings were reported along with the corresponding standard errors. Cronbach’s alpha was used to evaluate overall instrument- and factor-specific reliability. For Cronbach’s alpha, a reliability of at least 0.6 was considered acceptable, while the desired value was 0.7 or greater. The following fit indices were evaluated based on Hu and Bentler’s recommendations [20]: (1) maximum likelihood-based standardized root mean squared residual (SRMR, desired value 0.08 or less, indicating good fit), (2) comparative fit index (CFI, desired value 0.95 or greater), and (3) root mean square error of approximation (RMSEA, desired value 0.06 or less, acceptable value 0.08 or less), along with corresponding 95% confidence intervals. Factor (subscale) scores were summarized in terms of means and standard deviations. The analyses were conducted for the overall study population and stratified by discipline.

## 3. Results

### 3.1. Translation

The linguistic correspondence between SPICE-R2 and SPICE-R2F is available in Table 1. Regarding the word “discipline” in English, we used different words in French, depending on the context. For Q1 (question 1) we chose to translate it as “sector.” This vocabulary is used especially at the beginning of students’ courses of study. There is no equivalent word in English for the French word *filière* but “sector” was the best choice and was validated, as it associates a specific academic health specialization (inside the medicine or pharmacy curriculum) leading to a professional sector. Therefore, it made sense for the French students and health-care professionals. For Q4, however, we translated it as “training.” We took the definition of the discipline from the Larousse official French dictionary: “*Branch of knowledge that can provide material for teaching.*” Thus, several disciplines are included in the same training. Through training, we highlighted the different health professions. Conversely, we kept “discipline” for Q10. We emphasized that during training, there are different paths of specialization, involving different disciplines. Their uses in the French language are similar and do not interfere with the understanding of the sentences. For Q6, we did not literally translate “treated.” We opted for “receive a care.” This was decided to be in agreement with the meaning of “care” in the sentence. For Q7, we made a difference between “be trained to” and “be educated to.” In France, education can have a moral connotation added to the primary meaning of the term. The French Larousse gives a definition in this sense: “*Knowledge and practice of good manners, of the customs of society; good manners*” [21]. We wanted to exclude this perception by expressing “being trained.” The latter does not carry a moral intention. For Q9, we translated the notion of “patient/client-centeredness” to “the central place of the patient.” We removed the term “client.” In France, it is not in the medical culture to use the word “client” to designate a person needing health care. It is part of the lexical field of commerce. At the early stage of initial training, this term is not usual, and may be perceived as inappropriate by medical and pharmacy students.

### 3.2. Psychometric Validation of the Translation

#### 3.2.1. Descriptive Statistics

We collected 901 responses. This response rate was low compared to the potential of students solicited by the project. The University of Montpellier had a participation rate of around 31%, the University of Strasbourg 5.9%, the University of Paris 2.9%, and the University of Bordeaux 0.9% of those surveyed. The descriptive statistics of the sample of students who responded are given in Table 2. Over half (65%) the responses came from the University of Montpellier. The University of Paris had the second-highest participation. We obtained answers from all years of study. The 3rd, 4th and 5th years had higher rate of responses. These years correspond in the respective curricula to the start of hospital internships (3rd year in medicine and 5th year in pharmacy).

#### 3.2.2. Confirmatory Factor Analysis

The model fit for the CFA is summarized in Table 3. The model fit based on the RMSEA and SRMR criteria for the overall and each subpopulation was acceptable. An excellent model fit was observed when using the CFI. Overall, the results indicated adequate model fit of the SPICE-R2F.

Means of the total scores for the questions and by factor subsets are given in Table 4. The higher the score, the more the participants agreed with the items proposed. These are similar to the averages observed in the original study and in the German translation [17]. The subset of factor 1—Teamwork—seemed to have the strongest support from students compared to the other factors.

The standardized estimates of the loading coefficients for the latent variables are given in Table 5. They show that none of the latent variables (factor 1, 2, or 3) of the original population model [15] appeared adequate to describe the interindividual variability observed in the responses recorded. The answers to the questions, by factorial subset, appeared disparate, heterogeneous, and independent of one another. In the subset of factor 1 (Teamwork), the responses to items 4 and 10 are reliable, although insufficient to validate the factor as a whole. Item 4 exposes the need for participation in training experience with other students, while item 10 suggests the involvement of students in interprofessional projects. In these items, students were asked about their need for professional training, and not about their experience. Conversely, item 1 is factual and item 7 asks for the student’s opinion on other students’ training. In the subset of factor 3 (Outcome for the patient), item 9 seems reliable but insufficient to validate the factor as a relevant latent variable. This item corroborates the importance attached by the students to the management of the patient by an interprofessional team. Patient satisfaction (item 3) and economic benefits (item 6) are not recognized by French students as part of interprofessional collaborative practice.

To complete the standardized parameters of the overall study, the estimate by pharmacy (Table 6) and medicine (Table 7) sectors gives similar results.

Finally, the point estimators of the questionnaire’s Cronbach’s alpha are given in Table 8. These showed low internal consistency in the answers to the questions for each factor. Only factor 1 for medical students had acceptable reliability (0.7), and tended to suggest that the answers given to this factorial subset were consistent with one another.

## 4. Discussion

To our knowledge, our study is the first attempt to translate into French and validate a psychometric instrument to assess the representations of interprofessional education and practice among medical and pharmacy students in France.

We chose the physician and pharmacist student populations, as they demonstrated motivation for a collaborative project. These cohorts have the advantage of being well known by authors at both curriculum and interprofessional practice levels. They are also students for whom interprofessional training remains rare in France. The implementation of the SPICE-R2F tool on a population in which students’ representations have no benchmark of interprofessional training appeared to us as an opportunity to promote the enrichment of their university education.

To strengthen our results, we first ruled out any gap in translation that would explain a discrepancy in understanding between the English and French versions. The language level required to translate the instrument was not a hindrance for the team. The sentences were short and few. Following results recording and analysis, we had the translation reevaluated by several English speakers, different from those who worked initially on this project. They did not identify any difficulty or any potential misunderstandings related to the translation. The internal dispersion of the responses of French students and the lack of psychometric reliability of the latent variables established by the SPICE-R2 model led us to formulate several hypotheses. Above all, the main question is based on cultural differences between the USA and France, as well as the appropriation by the students of the concept of interprofessional health care in France.

The fit of the model to the data was adequate. In addition, we showed that the scores obtained among French students were similar, on average and by factorial subset, to the North American and German populations. Our translation of SPICE-R2F demonstrated a psychometric reliability to be improved on the cohort of French medicine and pharmacy students. The latent variables proposed in the model did not appear to be conclusive regarding the answers provided by the French students.

Our psychometric approach may have limits related to the quantitative approach of CFA. This is a method of analysis that makes it possible to measure a psychometric concept in an objective and standardized way, namely the interprofessional representations of students. As the team of Pudritz et al. [17] suggested, a qualitative approach would make it possible to explore the reasons underlying students’ representations of interprofessional education and training. Cronbach’s alpha coefficient, used to validate the consistency of the French responses, may also be discussed. Indeed, it is the most widely used psychometric fidelity index in educational sciences, and yet, according to several experts, it may underestimate fidelity results [22,23,24,25,26].

The electronic sending (email) of the questionnaire may bear limitations, since it showed an average response time of the students not allowing an in-depth reading of the explanatory documents of the approach. A framework for collecting data from students participating in interprofessional training should be discussed, including exchanges on social media, for example. Finally, despite a statistically satisfactory number of responses (n = 901), we surveyed only eight training and research units of the 61 existing in France. Therefore, the present proof of concept is therefore also intended to ultimately gather all the communities for a global evaluation in the 61 institutions.

We compared a country, France, with several states of the USA, possibly having two different epistemological approaches. The inconsistency of the internal responses to the questionnaire shows a different understanding of French medicine and pharmacy students of interprofessionalism. This finding corroborates those of Pudritz et al. The authors explained that one of the factors had low reliability due to the lack of interprofessional experience, although they did not survey the students on this point [17]. For Germany and France, interprofessional training is not included in common basic training. Collaborative health practice is still at an early stage and not very visible to the population. The role model in France of a collaborative practice is not easily accessible to students.

The cohort of students was spread over the entire curricula of medicine and pharmacy. We collected data over the 5 years of each curriculum. The students were enrolled from the 2nd to the 6th year of medicine and pharmacy studies. For the German team, they had targeted years at the end of the curricula. Making a comparison with North American students with a curriculum that includes this skill is perhaps an obstacle to the validation of a tool for a culture that does not have it. A cohort of 4th-year medical students and 5th-year pharmacy students may be more appropriate. These levels correspond to the years of entry to the hospital environment. This internship site allows an interprofessional experience for the students.

In France, the public health policy reforming the health-care system [27] shows, in certain aspects, significant delays when compared to American public health policy. Primary care organization is, for the most part, an isolated practice of medicine. With MSPs, we are at the beginning of a transition toward collaborative practice [28]. The USA showed examples of collaboration ahead of France. For example, in community pharmacies, there are medication therapy management (MTM) models that have been operational since 2003. These are targeted exchanges between patients and pharmacists in order to promote the effective use of medicines, opportunities for health-behavior change (i.e., lifestyle improvement), and interprofessional interventions and referrals to improve overall patient well-being. In MTM programs, a comprehensive medication review helps ensure that the patients have accurate medication lists. The community pharmacist is then able to discuss with the other health-care professionals on the interprofessional team the interventions and potential orientations resulting from these MTM visits [29]. A somewhat similar example in France are the shared medication reviews, made possible in community pharmacies since March 2018. Nonetheless, it should be noted that these reviews are still at an embryonic stage in French practices [30]. Despite these gaps, we see that collaborative practice is becoming an axis of development for public health. The major difference between the United States and France is the emphasis on IPE within the health professions’ education system. Indeed, in the USA, there is an accreditation system for medical and pharmacy degrees that does not exist in France [10,11]. The quality approach is constitutive of the education programs. It also has a role upstream and downstream of training. In addition, these approvals have a value for hiring and defining salaries. Accreditors rely on models, such as the Interprofessional Learning Continuum Model. This model makes it possible to ensure the quality of an interprofessional program. This approach contrasts with France. Beyond accreditation, interprofessional training is implemented in the curriculum. The spiral curriculum of the doctorate of medicine of the University of Chicago is an example among others. It is a depiction of the training program based on a preconceptualized increase in complexity [31]. It includes a module titled “Principles of Professionalism, Health Care, and Health Equity,” upon which “team-based care” is the basis for its phase 3. This phase 3, in the 4th year of medicine, corresponds to the preparation, teaching, and evaluation of the skills necessary for entry into “medical residency”—a transition to internship [32]. This integration of interprofessional training into medical and pharmacy degree programs contrasts with the French context, which remains at an early experimental stage [33].

In France, health training has an approach primarily based on knowledge rather than competence. An example that crystallizes this observation is the competition to access the third cycle of medical and pharmacy studies [34]. It focuses on learning theoretical knowledge rather than knowing how to act in complex health-care situations. Nevertheless, a change is taking place in medicine with the recent reform of the second cycle. It includes an evaluation of behavior and performance through simulation and objective structured clinical examination [35].

In such a context, it is necessary to collect traces of changes in students’ perceptions of interprofessional collaboration. Interprofessional teamwork improves population health, as outlined in the WHO framework [6]. Regarding our study, the physician–pharmacist collaboration was our driving force, particularly through the issues of drug iatrogenesis. The pharmacist stands out in this case as a key player, making it possible to link the prescriptions of different prescribers. However, its resources are still insufficiently mobilized in medical practice. This example illustrates a need for interprofessional physician–pharmacist training. However, to our knowledge, this does not really exist as a structured endeavor in our country. To improve and promote the development of such training, it is necessary to establish objective standardized measures on interprofessionalism. In the present study, we aimed to highlight the need in France for the development of health students’ adequate interprofessional representations, since their role in educating health-care students about their professional identities was clearly demonstrated [36].

Recommendations from the High Authority for Health [3] coupled with legislative action in the form of the law “Hospital, patients, health, and territories” [2] represent a major step forward in France. Unfortunately, this governmental action alone is not enough to transform health-care delivery toward a more interprofessional approach that leverages the knowledge and expertise of all French health-care professionals.

Collaboration and coordination across organizations that traditionally represent specific professions is needed. International models for such collaboration exist for possible replication and/or inspiration, including the Canadian Interprofessional Health Collaborative (CIHC) [37] and the US-based Interprofessional Education Collaborative (IPEC) [38]. Organizations such as CIHC and IPEC facilitated the development and/or adoption of consensus terminology and competence frameworks in their respective countries. As it relates to interprofessional collaborative clinical practice in France, shared language and educational targets/goals (i.e., competencies) will be of paramount importance for nationwide transformation. We suggest, based on the results of this study, that such transformation may begin via partnership in medicine and pharmacy.

We also recommend a continued focus on the development of valid and reliable instruments capable of measuring outcomes of interest and value in France. Many contemporary English-language instruments measure concepts and constructs elevated by the US-based National Academy of Medicine’s Interprofessional Learning Continuum Model [39]. It is possible, perhaps likely, that French authorities, experts in health-care delivery, and educational scholars may identify different constructs of interest based on the uniqueness and peculiarities of the French practice and educational systems. Such efforts will require the aforementioned collaboration and partnership of professional organizations in France.

## 5. Conclusions

The use of SPICE-R2F pointed out a probable different epistemological framework of interprofessional education and collaborative practice in France. Our results regarding the perceptions of interprofessionalism by medical and pharmacy students in France question their conceptualization of interprofessionalism. Do they lack collaborative practice role models? Does the absence of interprofessional education in their courses alter their understanding of a collaborative approach? These questions about our results led us to investigate interprofessional representations through a qualitative research approach, as suggested by Pudritz [17]. Improving French education regarding interprofessional practice appears as a clear need. In order to promote interprofessional education as well as a pedagogic approach based on scientific evidence, it is necessary to establish instruments for objective measurement of students’ learning and interprofessional competence. Representations of collaborative practice constitute a relevant evaluative approach.

Based on the present investigation, we plan to develop initial interprofessional education and training in France. The SPICE-R2F instrument has its place in the French-speaking educational evaluative arsenal, as it will also extend IPE and IPCP representations to the entire health-student community in addition to medicine and pharmacy students.

## Figures and Tables

**Table 1:** Comparison of the English (SPICE-R2) and French (SPICE-R2F) instruments. The elements appear in the order of the proposed factors: teamwork and team practice (factor 1), roles/responsibilities for collaborative practice (factor 2), and patient outcomes of collaborative practice (factor 3). In the survey, the items appeared chronologically according to their item number as presented in column 2.

Items	SPICE-R2	SPICE-R2F
Factor 1		
Q1	Working with students from different disciplines enhances my education.	Travailler avec des étudiants d’autres filières améliore ma formation.
Q4	Participating in educational experiences with students from different disciplines enhances my ability to work on an interprofessional team.	Participer à des expériences éducatives avec des étudiants issus d’autres formations améliore ma capacité future à travailler avec une équipe interprofessionnelle.
Q7	Health professional students from different disciplines should be educated to establish collaborative relationships with one another.	Les étudiants des professions de santé venant de différentes disciplines devraient être formés pour établir des relations de collaboration les uns avec les autres.
Q10	During their education, health professional students should be involved in teamwork with students from different disciplines in order to understand their respective roles.	Au cours de leur formation, les étudiants des professions de santé devraient être Impliqués dans du travail en équipe avec des étudiants venant de différentes disciplines afin de comprendre leurs rôles respectifs.
Factor 2		
Q2	My role within an interprofessional team is clearly defined.	Mon rôle au sein d’une équipe interprofessionnelle est clairement défini.
Q5	I have an understanding of the courses taken by, and training requirements of, other health professionals.	J’ai une compréhension des cours suivis par les autres professionnels de santé, ainsi que des exigences de leurs formations.
Q8	I understand the roles of other health professionals within an interprofessional team.	Je comprends le rôle des autres professionnels au sein d’une équipe interprofessionnelle.
Factor 3		
Q3	Patient/client satisfaction is improved when care is delivered by an interprofessional team.	La satisfaction des patients est améliorée quand ils/elles reçoivent des soins dispensés par une équipe interprofessionnelle.
Q6	Health-care costs are reduced when patients/clients are treated by an interprofessional team.	Les coûts des soins de santé sont réduits quand les patients reçoivent des soins par une équipe interprofessionnelle.
Q9	Patient/client-centeredness increases when care is delivered by an interprofessional team.	La place centrale du patient dans le soin est renforcée quand il est dispensé par une équipe interprofessionnelle.

**Table 2:** Demographics of study participants (n = 901).

Data Type	Number of Participants (%)
Curriculum	
Pharmacy	617 (69)
Medicine	284 (31)
University	
Montpellier	583 (65)
Paris	195 (22)
Strasbourg	101 (11)
Bordeaux	22 (2)
Year of study	
4	252 (28)
5	227 (25)
3	188 (21)
2	129 (14)
6	105 (12)

**Table 3:** Diagnostic adjustment criteria for SPICE-R2F.

	N	RMSEA	CFI	SRMR
Total	901	0.038 (95% CI: 0.027-0.050)	0.976	0.040
Pharmacy	617	0.037 (95% CI: 0.022-0.052)	0.973	0.046
Medicine	284	0.0046 (95% CI: 0.020-0.069)	0.967	0.060

**Table 4:** Quantitative estimates of responses to SPICE-R2F.

		Factor 1		Factor 2		Factor 3		Total	
	N	Mean	Standard Deviation	Mean	Standard Deviation	Mean	Standard Deviation	Mean	Standard Deviation
Combined	901	17.8	2.1	10.5	2.0	12.2	1.7	40.5	3.9
Pharmacy	617	18.0	3.7	10.7	1.9	12.4	1.7	41.2	3.7
Medicine	284	17.2	2.2	9.8	2.1	11.8	1.6	38.8	3.8

**Table 5:** Standardized estimators of the loading coefficients of the latent variables.

	Item	Estimation	Standard Deviation	*p*-Value
Factor 1	Q1	0.555	0.033	<0.0001
	Q4	0.706	0.028	<0.0001
	Q7	0.553	0.028	<0.0001
	Q10	0.746	0.027	<0.0001
Factor 2	Q2	0.379	0.047	<0.0001
	Q5	0.526	0.045	<0.0001
	Q8	0.648	0.056	<0.0001
Factor 3	Q3	0.538	0.040	<0.0001
	Q6	0.419	0.041	<0.0001
	Q9	0.754	0.043	<0.0001

**Table 6:** Standardized estimators of latent variable saturation coefficients, pharmaceutical sector.

	Item	Estimation	Standard Deviation	*p*-Value
Factor 1	Q1	0.571	0.039	<0.0001
	Q4	0.662	0.038	<0.0001
	Q7	0.478	0.039	<0.0001
	Q10	0.751	0.039	<0.0001
Factor 2	Q2	0.354	0.055	<0.0001
	Q5	0.508	0.053	<0.0001
	Q8	0.667	0.072	<0.0001
Factor 3	Q3	0.574	0.052	<0.0001
	Q6	0.442	0.049	<0.0001
	Q9	0.783	0.053	<0.0001

**Table 7:** Standardized estimators of loading coefficients of latent variables, medicine sector.

	Item	Estimation	Standard Deviation	*p*-Value
Factor 1	Q1	0.533	0.058	<0.0001
	Q4	0.747	0.043	<0.0001
	Q7	0.620	0.042	<0.0001
	Q10	0.733	0.037	<0.0001
Factor 2	Q2	0.364	0.088	<0.0001
	Q5	0.410	0.087	<0.0001
	Q8	0.788	0.141	<0.0001
Factor 3	Q3	0.426	0.065	<0.0001
	Q6	0.360	0.079	<0.0001
	Q9	0.614	0.081	<0.0001

**Table 8:** Cronbach’s coefficients of the sample.

	N	Alpha Coefficient
Total	901	0.6 (total) 0.66 (factor 1) 0.45 (factor 2) 0.48 (factor 3)
Pharmacy	617	0.58 (total) 0.62 (factor 1) 0.44 (factor 2) 0.51 (factor 3)
Medecine	284	0.55 (total) 0.7 (factor 1) 0.43 (factor 2) 0.37 (factor 3)

## Data Availability

Not applicable.
